# The Coated Tongue

**Published:** 1907-08

**Authors:** 


### THE COATED TONGUE.

A coated tongue, says Rollin, can depend entirely on pathological processes in the mouth or nasopharynx. Aside from this, however, twenty to thirty. Pleurisy is most frequent in the spring and least in the fall. Pleurisy affects children under fifteen with equal frequency for boys and girls, but is four times as frequent in men as in women. per as follows: Pleurisy occurs at all ages, but most frequently from certain conclusions may be formed regarding the function of the stomach. In hyperacidity of the stomach one sees as a rule a dark-red, moist, clean tongue. With a lack of hydrochloric acid, however, there is a pale thick-coated tongue. Rollin explains that in hyperacacidity the blood is nourished above normal, circulation in the tongue is very vigorous, and shedding of the superficial epithelium proceeds very rapidly, whereas in an acidity there is an anaemia with the consequence that the circulation lacks force to cast off the epithelium. In spite of the many statements to the contrary it is the opinion of his author that the coated tongue may often be of considerable diagnostic import.—Berliner Klinische Kochenschrift, 1906, No. 18.

